# Supplementary material for: Changing epidemiology of nephrotic syndrome in Nigerian children: A cross-sectional study
Source: PLoS One. 2020 Sep 21;15(9):e0239300. doi: 10.1371/journal.pone.0239300 (PMC7505420; doi:10.1371/journal.pone.0239300)
Supplement: Attachment — Submitted filename: Response to Reviewers.docx [file pone.0239300.s002.docx]

Dear Editor,

Thank you for giving us the opportunity to have our manuscript reviewed for possible publication in your Journal. Again, we are grateful for the useful feedback that have enriched the message of the manuscript.

We have carefully read all the comments provided and have revised the manuscript accordingly. Below is a point-by-point response to the comments raised during the peer review:
When submitting your revision, we need you to address these additional requirements.

**Editor:** Please ensure that your manuscript meets PLOS ONE's style requirements, including those for file naming. The PLOS ONE style templates can be found at

<https://journals.plos.org/plosone/s/file?id=wjVg/PLOSOne_formatting_sample_main_body.pdf> and

<https://journals.plos.org/plosone/s/file?id=ba62/PLOSOne_formatting_sample_title_authors_affiliations.pdf>

**Response**: We have substantially followed the Journal’s style requirements in the revised manuscript. Specifically, we have revised the font sizes for the various heading levels, removed funding source from acknowledge section, inserted the table and figures immediately after the paragraph where they are first mentioned and renamed the supplementary file.

**Editor:** For studies involving humans categorized by race/ethnicity, age, disease/disabilities, religion, sex/gender, sexual orientation, or other socially constructed groupings, authors should:
1) Explicitly describe their methods of categorizing human populations,
2) Define categories in as much detail as the study protocol allows,
3) Justify their choices of definitions and categories,
4) Explain whether (and if so, how) they controlled for confounding variables such as socioeconomic status, nutrition, environmental exposures, or similar factors in their analysis, and
5) Update outmoded terms and potentially stigmatizing labels to more current, acceptable terminology.
Examples: “Caucasian” should be changed to “white” or “of [Western] European descent” (as appropriate); “XXX victims” should be changed to “patients with XXX.”

**Response**: “Caucasian” has been replaced everywhere it appeared in the manuscript with “children of European descent” or “children with European ancestry”

**Editor:** In your ethics statement in the Methods section and in the online submission form, please provide additional information about the data used in your study. Specifically, please ensure that you have discussed whether all data were fully anonymized before you accessed them and/or whether the IRB or ethics committee waived the requirement for informed consent.
For the children also participating in the genetic study, please clarify whether written informed consent was obtained from parents or guardians of the children.

**Response**: data for about 67% of the children included in this report were collected as part of their participation in a genetic study on childhood nephrotic syndrome (genetic analysis is still ongoing). For these children, their caregivers provided written informed consent; children older than 12 years also provided assent. For the other 1/3^rd^ of the participants included in this study, the requirement for consent was waived because (1.) only information recorded in their medical records as part of routine patient care was assessed, and (2.) the vast majority of these patients were no longer in active follow up either because the family has moved out of town, nephrotic syndrome is cured or child had died. Furthermore, all retrieved medical information was anonymised to maintain confidentiality. The ethic statement in the manuscript has been improved upon to provide this clarity.

The formal name of the Ethics committees are: (1.) LUTH Health Research Ethics Committee and (2.) LASUTH Health Research Ethics Committee

**Editor:** In figures 2 and 3, please indicate your measures of significance either with symbols or with your actual p-values.

**Response:** The p values have been added to the two figures

Additional Editor Comments (if provided):

The reviewers have raised concerns that need to be addressed before the article can be considered for publication.

[Note: HTML markup is below. Please do not edit.]

Reviewers' comments:

Reviewer's Responses to Questions

**Comments to the Author**

1. Is the manuscript technically sound, and do the data support the conclusions?

The manuscript must describe a technically sound piece of scientific research with data that supports the conclusions. Experiments must have been conducted rigorously, with appropriate controls, replication, and sample sizes. The conclusions must be drawn appropriately based on the data presented.

Reviewer #1: Partly

Reviewer #2: Partly

2. Has the statistical analysis been performed appropriately and rigorously?

Reviewer #1: Yes

Reviewer #2: I Don't Know

3. Have the authors made all data underlying the findings in their manuscript fully available?

The [PLOS Data policy](http://www.plosone.org/static/policies.action#sharing) requires authors to make all data underlying the findings described in their manuscript fully available without restriction, with rare exception (please refer to the Data Availability Statement in the manuscript PDF file). The data should be provided as part of the manuscript or its supporting information, or deposited to a public repository. For example, in addition to summary statistics, the data points behind means, medians and variance measures should be available. If there are restrictions on publicly sharing data—e.g. participant privacy or use of data from a third party—those must be specified.

Reviewer #1: Yes

Reviewer #2: Yes

4. Is the manuscript presented in an intelligible fashion and written in standard English?

PLOS ONE does not copyedit accepted manuscripts, so the language in submitted articles must be clear, correct, and unambiguous. Any typographical or grammatical errors should be corrected at revision, so please note any specific errors here.

Reviewer #1: Yes

Reviewer #2: Yes

5. Review Comments to the Author

Please use the space provided to explain your answers to the questions above. You may also include additional comments for the author, including concerns about dual publication, research ethics, or publication ethics. (Please upload your review as an attachment if it exceeds 20,000 characters)

**Reviewer #1**: My reason for suggesting a minor revision rests mainly in how the authors calculated the prevalence of the AKI.You have partly answered this under "Limitations" - see lines 242 to 246 of manuscript. What I find disappointing, is that urine output, urine microscopy, FeNa nor urinary Beta2microglobulin / creatinine ratios were not looked at to substantiate possible AKI .The back calculated serum creatinine would tend to increase the prevalence of AKI, particularly with the the GFR set at 120 ml/min/1.73m2 ie the basal serum creatinine would then be artificially lower, and therefore a 1.5 times increase would more easily be reached, giving an easier overestimate of AKI. Therefore, your estimate of 40.9% of children with AKI at the time of diagnosis, is likely to be an over-estimate. However, AKI affecting the tubules only, could be missed if one does not address the examination of the urine as given above.
I do realize that AKI has been documented at even higher levels, but my concerns above still apply. Please see references 23, 25 and 31 of your paper- AKI often aggravated in the ICU or with the use of nephrotoxic drugs.

**Response**: We agree with the comments of both reviewers that using back-calculation to determine serum creatinine may have substantial inherent weakness. As a result, we have removed all aspects of the manuscript detailing the methods, results and discussion of acute kidney injury. However, we still retained aspects of the manuscript that relates to serum creatinine and estimated glomerular filtration rate values

**Reviewer** #1: On the other hand, over the last seven years, steroid sensitive nephrotic syndrome in Black children in Lagos, particularly in the Yoruba ethnic group, is comparable to the rate in Asians and Caucasians elsewhere.I was surprised that over 80% of the steroid sensitive group were hypertensive. Was this particularly in the younger age group? Were the children quiet when blood pressure done? - adequate cuffs? - upper arm at heart level if child sitting? Was it aggravated by the steroid dosage?

**Response**: The correct reading of the results on Table 1 is: 27.5% (57) of the of 207 children with SSNS had elevated BP compared with 32.4% (11) of the 34 children with SRNS. This is so when the results are read down the columns rather than across the rows.

**Reviewer #2**: The questions regarding the paper are as follows:
1. Are all children int these areas access the teaching hospitals or there are private practises handling these children?

**Response**: there are several private hospitals in Lagos and they may have some children with nephrotic syndrome. However, two of the authors of these manuscripts (CIE and AUS) are the only active paediatric nephrologists in the city. Our experience is that children with difficult-to-treat nephrotic syndrome are more likely to be referred to our centres rather than managed in the other hospitals without a paediatric nephrologist
**Reviewer #2**. Was the cyclophosphamide given IVI or orally?

**Response**: Initially cyclophosphamide was being given as monthly intravenous dose but in the latter years that the study period covered, it was mostly administered orally
**Reviewer #2**. Were the levels of cyclosporin monitored as higher doses cause renal dysfunction due to nephrotoxicity?

**Response**: Yes, cyclosporine drug levels were monitored, but not more frequently than every 3 months after the first month of starting. The frequency of monitoring depended on ability of the family of the child to afford it

**Reviewer #2**. The back calculation of creatinine is not tested scientifically, the authors quoted for this method reference 25," However, this method of estimating baseline kidney function has not been validated, nor has it been systematically compared with other methods. Substantial interstudy heterogeneity also exists in estimating baseline SCr"
5. Thus my recommendation is to leave out the Acute renal failure objective. the rest of the conclusions are valid according to their study.

**Response**: We agree with this recommendation and have removed the aspect of AKI prevalence from all aspects of the manuscript.
